# Transcriptomic and functional network features of lung squamous cell carcinoma through integrative analysis of GEO and TCGA data

**DOI:** 10.1038/s41598-018-34160-w

**Published:** 2018-10-26

**Authors:** Yin Li, Jie Gu, Fengkai Xu, Qiaoliang Zhu, Di Ge, Chunlai Lu

**Affiliations:** Department of Thoracic Surgery, Zhongshan Hospital, Fudan University, Shanghai, P. R. China

## Abstract

Lung squamous cell carcinoma (LUSC) is associated with poor clinical prognosis and lacks available targeted therapy. Novel molecules are urgently required for the diagnosis and prognosis of LUSC. Here, we conducted our data mining analysis for LUSC by integrating the differentially expressed genes acquired from Gene Expression Omnibus (GEO) database by comparing tumor tissues versus normal tissues (GSE8569, GSE21933, GSE33479, GSE33532, GSE40275, GSE62113, GSE74706) into The Cancer Genome Atlas (TCGA) database which includes 502 tumors and 49 adjacent non-tumor lung tissues. We identified intersections of 129 genes (91 up-regulated and 38 down-regulated) between GEO data and TCGA data. Based on these genes, we conducted our downstream analysis including functional enrichment analysis, protein-protein interaction, competing endogenous RNA (ceRNA) network and survival analysis. This study may provide more insight into the transcriptomic and functional features of LUSC through integrative analysis of GEO and TCGA data and suggests therapeutic targets and biomarkers for LUSC.

## Introduction

Every year, nearly 1.8 million people are diagnosed with lung cancer^1,2^. Lung cancer has become the leading cancer cause of death and kills more people annually than colorectal, breast, prostate and pancreatic cancers combined^3^. Lung squamous cell carcinoma (LUSC) is a subtype of non-small cell cancer and accounts for approximately 40% of all lung cancer, which based on age or extent of tobacco exposure. LUSC is associated with poor clinical prognosis and lacks targeted agents available compared to lung adenocarcinoma^4,5^. The essential biomarkers and precise targets for the development and progression of LUSC remain unclear.

High throughput microarray platforms emerge as a promising and useful tool for detection of genetic alterations in carcinogenesis and discovering biomarkers for many diseases^6^. However, individual microarray investigation often shows a bias toward the identification of high-abundance molecules due to possession of insufficient numbers of specimens and therefore often fails^5^. By integrating multiple microarray datasets, we can provide sufficient samples and come up with more convincing results. However, the microarray technique itself has some drawbacks. An array can only detect sequences that the array was designed to identify and the relative concentration measurement is relative indirect^7^. Nevertheless, with the revolution of genome technologies, next-generation sequencing (NGS) is on the stage^8^. Sequencing is independent on previous knowledge of which nucleic acids may be present and sequencing can also independently detect closely related gene sequences. Therefore, identification of high-abundance molecules would become much more reliable via integrating the differentially expressed genes derived from multiple microarray datasets analysis with sequence-based data.

Furthermore, joint analysis of the array-based and sequence-based data of LUSC maybe a novel analytical strategy. In our present study, we conducted our data mining analysis for LUSC by integrating the differentially expressed genes acquired from Gene Expression Omnibus (GEO) database into The Cancer Genome Atlas (TCGA) database. As a result, we discovered some co-differentially expressed genes in LUSC. Based on these genes, we performed a series of analyses including functional enrichment analysis, protein-protein interaction analysis, survival analysis, construction of competing endogenous RNA network. We discovered some new biomarkers that have never been thought to be involved in LUSC. Our study could provide more insights into the molecular mechanism of this prevalent and devastating disease.

## Materials and Methods

### Microarray studies, data sets and clinical sample characteristics from GEO data repository

**Figure 1:**
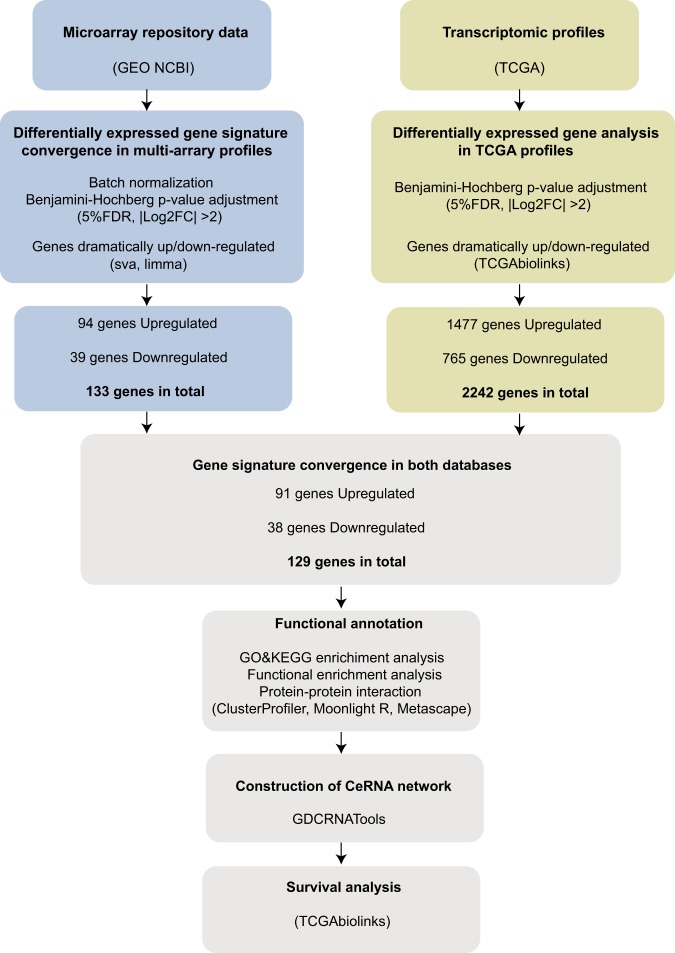
Flowchart for bioinformatics analysis of publicly available data from both GEO and TCGA databases.

Gene Expression Omnibus (GEO), NCBI’s publicly available genomics database, which collects submitted high throughput gene expression data, was thoroughly queried for all datasets involving studies of LUSC. Studies were considered eligible for our following analysis according to the following criteria: (1) Studies with squamous cell carcinoma tissue samples. (2) Studies with information about the technology and platform utilized for studies. (3) Studies with the presence of normal groups as the control. Based on these criteria, seven datasets for LUSC were downloaded from the repository. Principal component analysis (PCA) was done for the datasets for dimensionality reduction and quality control. If the quality of a particular sample is not good enough, it would be excluded for subsequent analysis. Details of each microarray study, including sample descriptions are provided in Table 1. Our workflow for bioinformatics analysis of publicly available datasets from both GEO and TCGA databases is illustrated in Fig. 1.

**Table 1:** Details of LUSC studies and associated microarray datasets from GEO database.

GSE	Publication	Total differentially expressed genes	Up-regulated	Down-regulated	Technology/Platform	Sample size for each group	Age	Sex (M:F)
GSE8569	Journal of pathology	87	50	37	CNIO Human Oncochip 2.0	tumor:35; adjacent normal tissue:6	not provided	All male
GSE21933	BMC Cancer	1222	524	698	Phalanx Human OneArray	tumor:10; adjacent normal tissue:10	73, 65, 74, 71, 62, 67, 75, 77, 67, 81	M:10
GSE33479		983	431	552	Agilent-014850 Whole Human Genome Microarray 4x44K G4112F (Probe Name version)	tumor:14; adjacent normal tissue:13	75, 67, 55, 75, 64, 55, 70, 65, 68, 44, 72, 52, 66, 56	M:F 9:5
GSE33479	Journal of Bioinformatics Research Studies	1037	427	610	[HG-U133_Plus_2] Affymetrix Human Genome U133 Plus 2.0 Array	tumor:16; adjacent normal tissue:4	64, 62, 62, 58	M:4
GSE40275	Molecular cancer research	1153	654	499	Human Exon 1.0 ST Array [CDF: Brainarray Version 9.0.1, HsEx10stv2_Hs_REFSEQ]	tumor:5; adjacent normal tissue:14	38, 59, 65, 78, 80	M:F 4:1
GSE62113	Nature Communications	552	273	279	Illumina HumanHT-12 WG-DASL V4.0 R2 expression beadchip	tumor:2; adjacent normal tissue:6	not provided	not provided
GSE74706	Cancer research	1753	720	1033	Agilent-026652 Whole Human Genome Microarray 4x44K v2	tumor:8; adjacent normal tissue:8	not provided	not provided
TCGA		2242	1477	765	Illumina HiSeq		see Table 2

**Table 2:** The clinical information and sample size for TCGA LUSC dataset.

	Alive (n = 343)	Dead (n = 161)	Total (n = 504)	P Value
**Gender**				
Female	90 (26.2%)	41 (25.5%)	131 (26.0%)	
Male	253 (73.8%)	120 (74.5%)	373 (74.0%)	0.94
**Age**				
Mean (SD)	66.6 (8.5)	68.7 (8.6)	67.3 (8.6)	
Median [Min, Max]	68 [39, 84]	70 [40, 90]	68 [39, 90]	
**Race**				
Asian	6 (2.2%)	3 (2.4%)	9 (2.3%)	
Black Or African American	14 (5.2%)	17 (13.8%)	31 (7.9%)	
White	248 (92.5%)	103 (83.7%)	351 (89.8%)	0.013
**Stage**				
Stage IA	69 (25.7%)	21 (17.1%)	90 (23.0%)	
Stage IB	100 (37.3%)	52 (42.3%)	152 (38.9%)	
Stage II	1 (0.4%)	2 (1.6%)	3 (0.\%)	
Stage IIA	53 (19.8%)	12 (9.8%)	65 (16.6%)	
Stage IIB	65 (24.3%)	30 (24.4%)	95 (24.3%)	
Stage IIIA	40 (14.9%)	23 (18.7%)	63 (16.1%)	
Stage IIIB	9 (3.4%)	10 (8.1%)	19 (4.9%)	
Stage IV	4 (1.5%)	3 (2.4%)	7 (1.8%)	
Stage I		3 (2.4%)	3 (0.8%)	
Stage III		3 (2.4%)	3 (0.8%)	0.095

### Differential expression analysis

Heterogeneity and potential variables are commonly recognized as major sources of bias and variability in high-throughput experiments. Since the datasets we recruited for our multi-datasets analysis were based on different platforms and samples were handled on different days, in different groups or by different people. Besides, datasets GSE40275 and GSE61223 only have 5 and 2 tumor samples respectively and using few samples can affect the performance of statistical analysis and provides unreliable results. Therefore, we first integrated all samples of seven datasets to significantly improve the number of samples (61 normal samples vs. 88 tumor samples) so as to avoid generating less reliable results followed by batch normalization in the R computing environment using sva package^9^. The unnormalized raw data was summarized as the form of the matrix and can be acquired in Supplementary Table 1. Next, we performed the differential analysis (|Log_2_FC| > 2, adjusted p-value < 0.05) by comparing tumor tissues to normal tissues in the R computing environment using limma package^10^.

### Integration of the differentially expressed genes in TCGA database

The Cancer Genome Atlas (TCGA), a project supported by the National Cancer Institute (NCI) and National Human Genome Research Institute (NHGRI), has generated comprehensive, multi-dimensional maps of the key genomic changes in various types of cancers. In order to obtain a consensus of differentially expressed genes, gene expression quantification data and clinical information of LUSC patients in TCGA database were downloaded using TCGAbiolinks^11^. All data were normalized and processed with TCGAbiolinks pipeline. The TCGAbiolinks principle of differential analysis is to first convert the count matrix into an edgeR object^12^, then each gene gets assigned the same dispersion estimate, then performs pair-wise tests for differential expression between two groups, and finally takes the output using the False Discovery Rate (FDR) correction, and returns the top differentially expressed genes^11^. The parameters set for differential expression analysis were FDR < 0.05 with |Log_2_FC| > 2. Subsequently, we combined the differentially expressed genes acquired from GEO and TCGA databases to get the convergence gene signatures.

### Circular visualization of the consensus differentially expressed genes

To help us have a better view of consensus differentially expressed genes including their symbols and chromosomal locations. Circos (http://circos.ca/) was used for our data presenting^13^.

### GO and KEGG pathway analysis, functional enrichment analysis, and protein-protein interaction

Gene ontology (GO), KEGG pathway enrichment analyses were performed in R using the function of clusterProfiler^14^. Functional enrichment analysis was performed using the latest version of moonlightR (FDR < 0.05, Moonlight z-score > 1)^15^. Protein-protein interaction analysis was performed to using Metascape (http://metascape.org).

### Construction of ceRNA network

To find out whether these 129 genes exist competing endogenous regulating network mediated by long non-coding RNAs (lncRNAs) and micro RNAs (miRNAs). A competing endogenous RNA (ceRNA) network was built using GDCRNATools^16^. The major criteria of building ceRNA network in GDCRNATools are: (1) The lncRNA and mRNA must share a significant number of miRNAs. (2) Expression of lncRNA and mRNA should be positively related. (3) miRNAs should play similar roles in regulating the expression of lncRNA and mRNA. We followed the pipeline of GDCRNATools to first identify differentially expressed lncRNAs (DElncRNAs) and differentially expressed miRNAs (DEmiRNAs) using the edgeR^12^ method (FDR < 0.05 with |Log_2_FC| > 2). Next, we used the function of GDCRNATools to construct the network, total read counts for 5p and 3p strands of DEmiRNAs were obtained from isoform quantification files, miRcode was used to collect predicted and experimentally validated lncRNA targets^17^. StarBase v2.0 was used to predict miRNA-mRNA interactions^18^. Visualization of the ceRNA was performed by Cytoscape^19^.

### Survival analysis

To see whether these 129 genes and DElncRNAs were related to prognostic significance, survival analysis was performed in the R environment using TCGAbiolinks^11^. We used clinical information to plot the survival curves for 1/3 of patients with higher expression of a specific gene versus the 1/3 of patients with lower expression of this gene (p < 0.05).

## Results

### Principal component analysis verifying independence of each group

**Figure 2:**
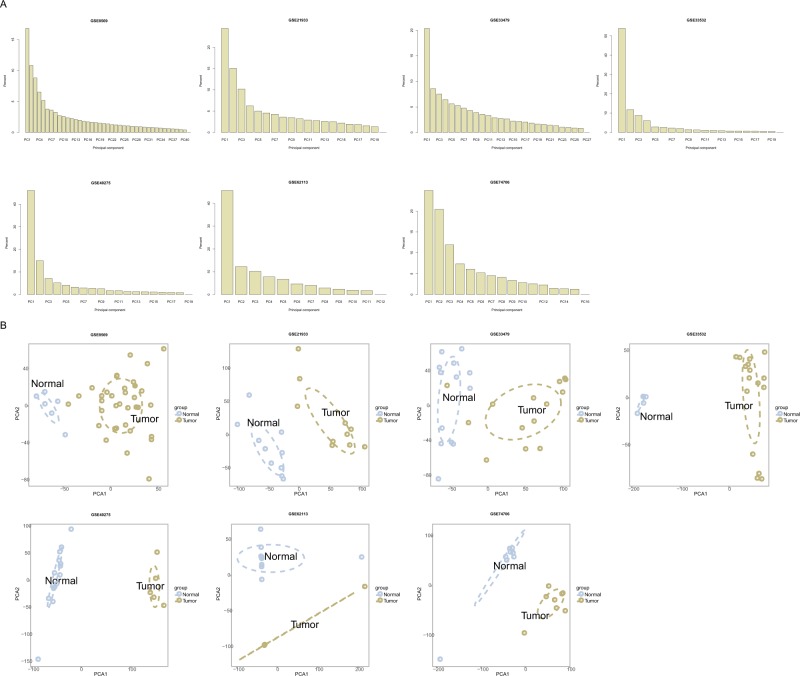
Results from the principal component analysis for microarray studies downloaded from the GEO database. (**A**) Bar plots showing the proportion of variance evaluated for each of the five microarray datasets. (**B**) Two-dimensional plots of normal and tumor groups with the top two principal components. Horizontal and vertical axes represent the distribution of each sample within PCA1 and PCA2 respectively. PCA1: principle component 1; PCA2: principal component 2.

To distinguish the significant difference between normal and tumor samples of GEO data, PCA was performed to reduce the dimensionality and evaluate the independence of each group. The results showed that normal samples vs. tumor samples in the datasets (GSE8569, GSE21933, GSE33532, GSE40275, GSE62113, GSE74706) displayed a significant difference except for dataset GSE33479, whose two tumor samples GSM828337 and GSM828345 were close to normal samples, so we removed these two samples for the subsequent analysis (Fig. 2B). The contribution rate for each principal component is on the vertical axis (Fig. 2A). The cumulative contribution rates of the PC1 and PC2 of each of the seven datasets are 27.64%, 39.50%, 28.94%, 65.74%, 61.05%, 57.85% and 45.44% respectively. The horizontal axis stands for the number of principal components required to reach a cumulative proportion of 100%. It was obvious that the first two components were enough to separate the two groups, indicating each group is independent of each other (Fig. 2B).

### Convergence of gene expression signatures across different studies of LUSC

**Figure 3:**
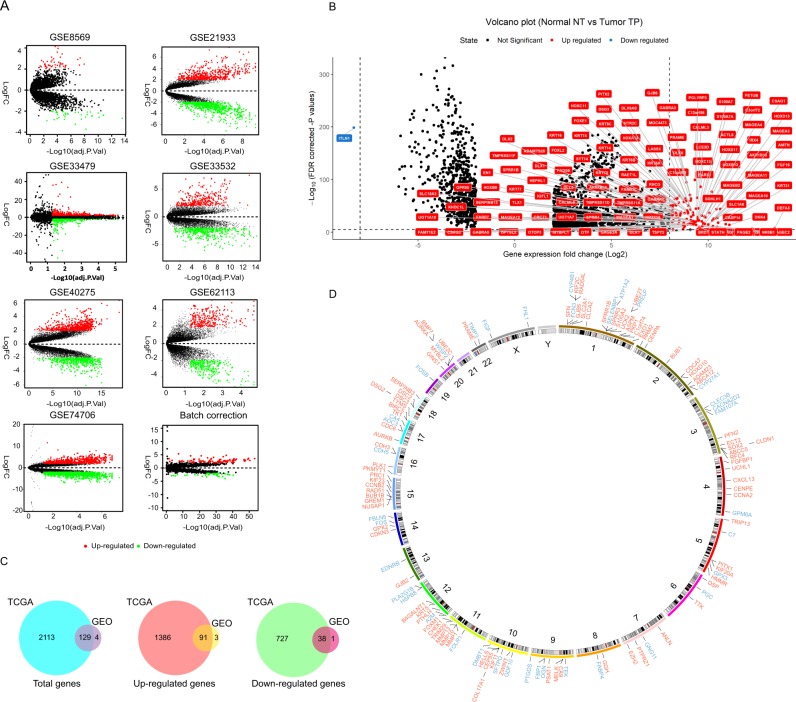
Convergence of gene expression signatures across different studies of LUSC. (**A**) Volcano plots showed the number of differentially expressed genes identified from each of the seven GEO datasets and after batch correction. (**B**) Volcano plot showed the number of differentially expressed genes in TCGA. (**C**) Venn diagram demonstrates the intersections of genes between GEO data and TCGA data. (**D**) Chromosome mapping of consensus genes.

Data integration is becoming increasingly necessary to investigate the complex genetic mechanisms by applying appropriate statistical method^20^. As the outputs of individual experiments can be rather noisy, it is essential to look for findings that are supported by several pieces of evidence to increase the signal and lessen the fraction of false positive findings. We used batch correction to reduce variability and then used limma package^10^ (|Log_2_FC| > 2, adjusted P value < 0.05) to identify differentially expressed genes. Table 1 demonstrates the number of differentially expressed genes identified from each of the seven datasets and TCGA data. Volcano plots in Fig. 3A showed the number of differentially expressed genes identified from each of the seven datasets and the number of differentially expressed genes after batch correction. We found 94 up-regulated genes and 39 down-regulated genes after batch normalization (Fig. 3A). For TCGA data, we found a total of 2242 differentially expressed genes with 1477 of them up-regulated and 765 genes down-regulated. Here, we demonstrate the names of genes with |Log_2_FC| > 8 (Fig. 3B). Venn diagram demonstrates the intersections of genes between GEO data and TCGA data, and 129 co-differentially expressed genes (91 up-regulated and 38 down-regulated) were found (Fig. 3C). Chromosome mapping of consensus genes revealed chromosome distribution, with chromosomes 1 containing the greatest number of dysregulated genes in LUSC (Fig. 3D). Interestingly, while two genes on the X chromosome showed dysregulation in LUSC (FHL1 and FIGF), not a single Y chromosome gene was affected.

**Figure 4:**
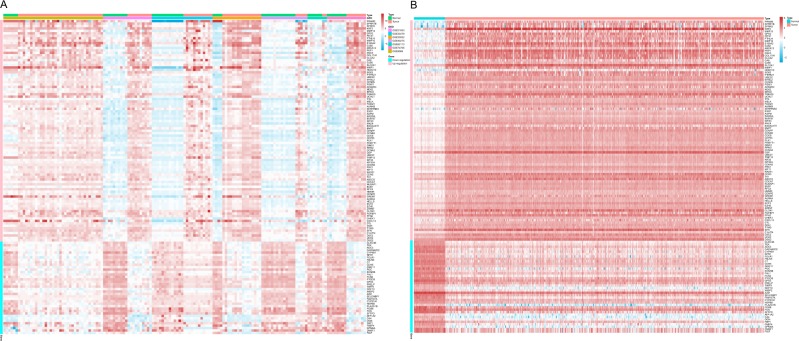
The expression changes of these genes in GEO and TCGA data. (**A**) Heatmap of differentially expressed genes in GEO dataset coloring the samples-groups. (**B**) Heatmap of differentially expressed genes in TCGA dataset coloring the groups.

In Fig. 4A,B we displayed the expression changes of these genes in GEO and TCGA data, respectively. More information including the fold change and FDR of these 129 genes can be found in Supplementary Table 2. These 129 genes were further subjected to functional annotation and protein to protein interaction analysis to determine the biological significance of this cross-study convergence in the pathogenesis of LUSC.

### GO terms and KEGG pathway analysis, functional enrichment analysis and protein-protein interaction

**Figure 5:**
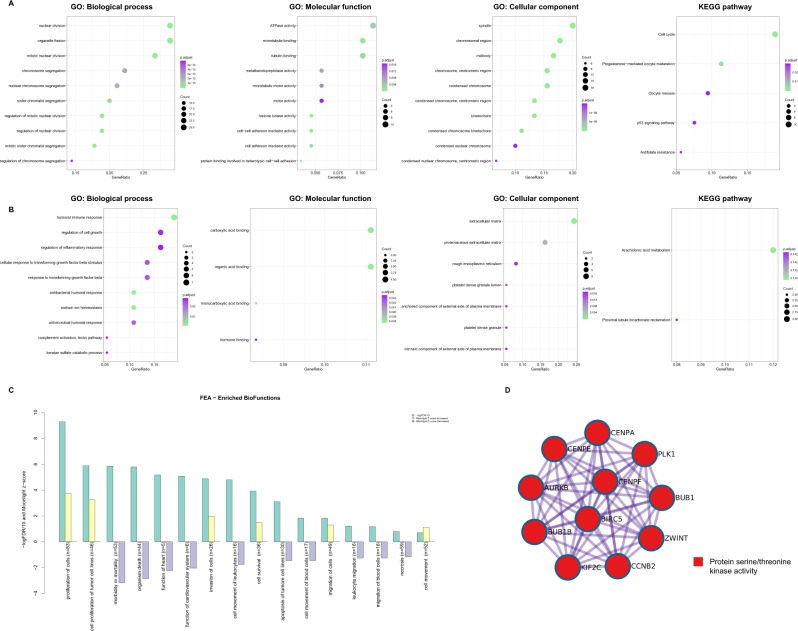
GO annotations, KEGG pathways, functional enrichment analysis and protein-protein interaction of up-regulated gene and down-regulated genes in LUSC. (**A**) The bubble plots showing GO and KEGG pathway enrichment data for genes that were up-regulated. (**B**) The bubble plots showing GO and KEGG pathway enrichment data for genes that were down-regulated. (**C**) Functional enrichment analysis plot. A negative z-score indicates that the activity is decreased. A positive z-score indicates that the activity is increased. (**D**) Protein-protein interaction network.

To explore the potential biological functions of the consensus genes, GO terms, KEGG pathway and functional enrichment analyses were performed. The GO annotation results have three parts: biological process, molecular function, and cellular component. The results revealed that the biological processes and molecular functions primarily associated with the up-regulated genes were nuclear division, organelle fission, mitotic nuclear division, ATPase activity, microtubule binding, and tubulin binding. Besides, these up-regulated genes were also strongly associated with cellular components of spindle, chromosomal region and midbody. The KEGG pathway analysis showed the up-regulated genes were significantly enriched in cell cycle, progesterone−mediated oocyte maturation, oocyte meiosis, and p53 signaling pathway (Fig. 5A). For down-regulated genes, humoral immune response, regulation of inflammatory response, regulation of cell growth, response to transforming growth factor beta, and carboxylic acid binding were found to be dominant biological processes and molecular functions. For cellular components, these down-regulated genes were mainly associated with extracellular matrix, proteinaceous extracellular matrix, and rough endoplasmic reticulum. The arachidonic acid metabolism and proximal tubule bicarbonate reclamation were pathways associated with the down-regulated genes (Fig. 5B). The complete results of GO and KEGG analyses can be found in Supplementary Table 3. Functional enrichment analysis indicated that expression changes of these genes could lead to increased activities of proliferation of cells, cell proliferation of tumor cell lines, invasion of cells, cell survival, migration of cells and cell movement in LUSC and decreased activities of organism death, cell movement of leukocytes, apoptosis of tumor cell lines, cell movement of blood cells, leukocyte migration, migration of blood cells and necrosis. All these functions are critically important in tumor cell survival, invasion and immune escape (Fig. 5C). Specific data of functional enrichment analysis can be found in Supplementary Table 4. Figure 5D showed the protein-protein interaction network. PPI enrichment analysis was done with the following databases: BioGrid16^21^, inWeb_IM17^22^ and OmniPath18^23^. Molecular Complex Detection (MCODE) algorithm^24^ was further applied to identify densely connected network components if there are more than two proteins in a network. We found that CCNB2, PLK1, KIF2C, CENPA, CENPF, BUB1, BUB1B, BIRC5, CENPE, ZWINT, AURKB, CHEK1, EXO1, RAD51, and RFC4 can interact with each other and this interaction was predominantly associated with protein serine/threonine kinase activity.

### ceRNA network

**Figure 6:**
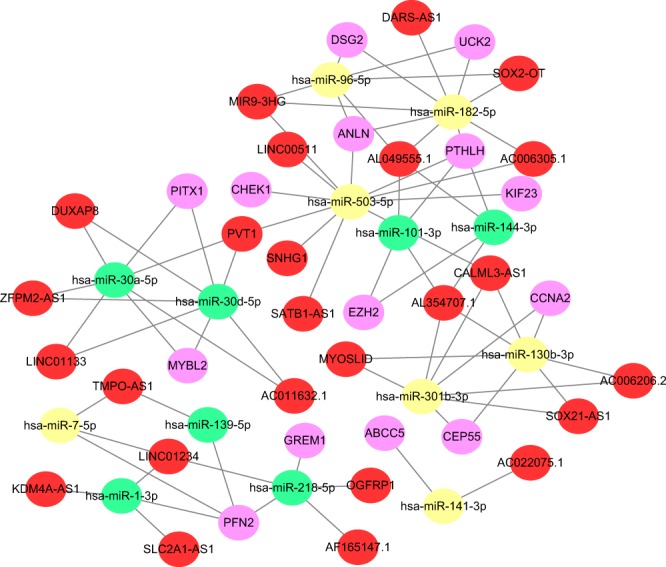
CeRNA network. LncRNA–miRNA–mRNA interactions in LUSC. Red indicates up-regulated lncRNAs, purple indicates up-regulated mRNAs, yellow stands for over-expressed miRNAs, and green means down-regulated miRNAs.

By using GDCRNAtools, a total of 124 DElncRNAs (|Log_2_FC| > 2, FDR < 0.05) and 74 DEmiRNAs (|Log_2_FC| > 2, FDR < 0.05) were found to exhibit a significant difference in LUSC compared with control (Supplementary Table 5). Next, lncRNA-miRNA interaction was predicted based on miRcode^17^ and miRNA-mRNA interaction was collected based on starBase v2.0^18^. CeRNA network was visualized using Cytoscape software. 25 lncRNAs, 14 miRNAs and 14 mRNAs (PTHLH, EZH2, CEP55, CCNA2, PFN2, ABCC5, ANLN, UCK2, DSG2, GREM1, MYBL2, PITX1, CHEK1, KIF23) were included in the network (Fig. 6). Red indicates up-regulated lncRNAs, purple indicates up-regulated mRNAs, yellow stands for up-regulated miRNAs, and green means down-regulated miRNAs. Interestingly, all lncRNAs and mRNAs were up-regulated. Specific information of ceRNA network is in Supplementary Table 6.

### Survival analysis

**Figure 7:**
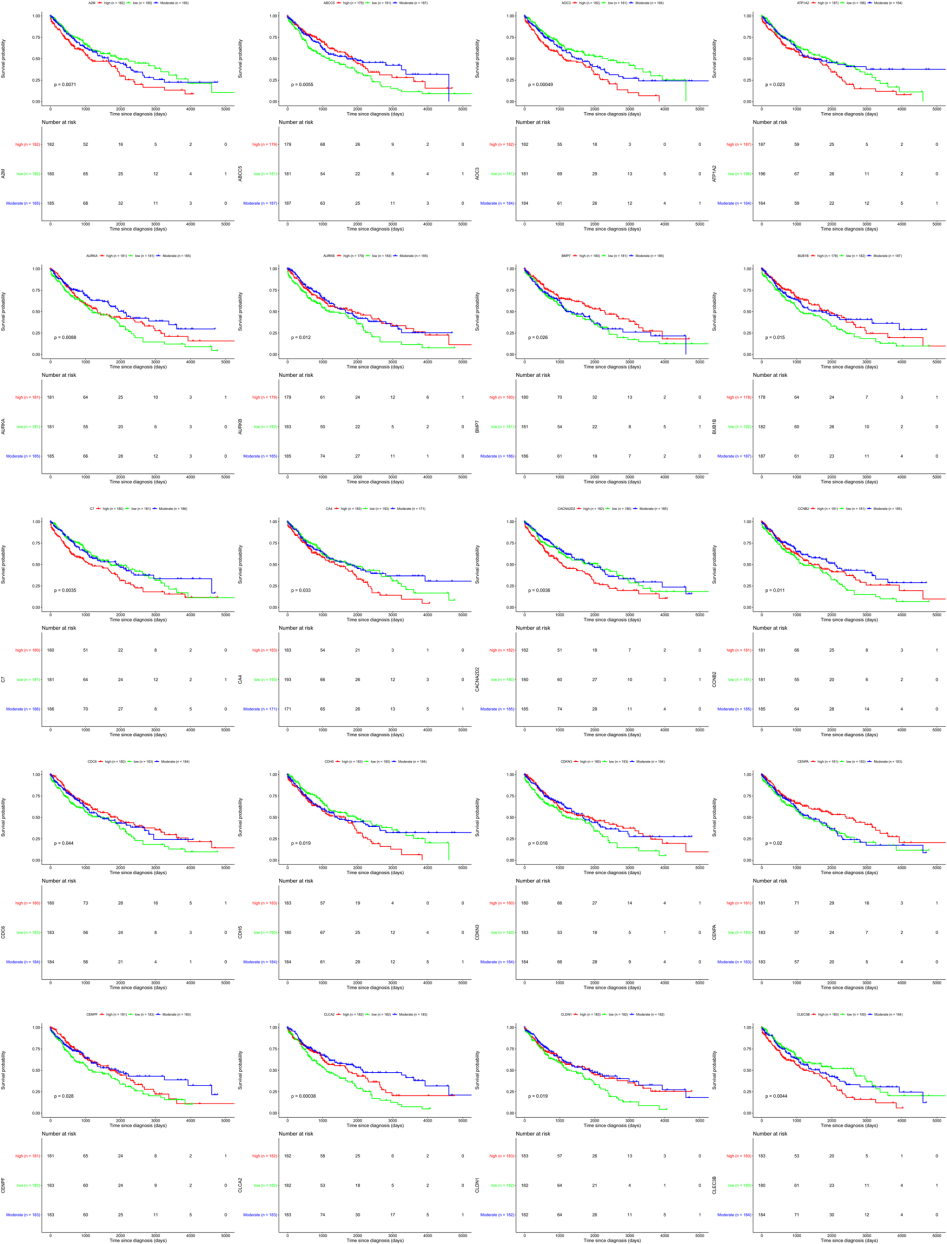
Survival analysis for differentially expressed genes in LUSC. Survival curves showing 20 examples of genes which were related to overall patient survival rate. P-value set for this analysis is less than 0.05.

Base on TCGA data and clinical information, we analyzed the survival curves for patients by comparing 1/3 of patients with higher expression of a certain gene to 1/3 of patients with lower expression. Of the 129 genes, we found that 60 genes were statistically related to the overall survival rate (p < 0.05). Here, we exhibited 20 examples of these genes (Fig. 7), the remaining can be found in Supplementary Figs 1 and 2. Expression changes of these 60 genes can be obtained in Supplementary Table 7. For these 60 genes, EZH2, ABCC5, and KIF23 were in the ceRNA network and could be modulated by corresponding lncRNAs and miRNAs. EZH2, ABCC5, and KIF23 were up-regulated in LUSC and patients with low expression levels of these three genes had shorter survival times (Fig. 7, Supplementary Figs 1 and 2). We also found that LncRNAs KC6, PART1, SFTA1P, and SNHG1 were statistically related to the overall survival rate (Supplementary Fig. 3, p < 0.05). Functional enrichment analysis indicated that the 60 overall survival related-genes were involved in the cell proliferation of tumor cell lines, perinatal death, invasion of cells, organism death, proliferation of cells, neonatal death and migration of cells (Supplementary Fig. 4 and Table 8).

## Discussion

LUSC has been regarded as the “neglected sibling” compared with lung adenocarcinoma due to lack of effective targeted treatment options. The mutations of epidermal growth factor receptor (EGFR) kinase, as well as fusions in the anaplastic lymphoma kinase (ALK), lead to a dramatic change in the treatment of patients with lung adenocarcinoma^25–27^. Unfortunately, EGFR mutations and ALK fusions are typically not present in LUSC^28^, and novel targeted agents for adenocarcinoma of the lung ineffective against LUSC. So, new classes of biomarkers with high efficiency, high specificity, and high sensitivity are required as novel molecules for diagnosis and prognosis of LUSC.

Integrating multiple individual data has been showed to improve detection power^29^. Integration of multiple arrays is considered a better approach of enhancing the reliability of results than individual array analysis. PCA is a sophisticated technique widely used for reducing the dimensions of multivariate problems and evaluating independence without losing much information^30^. In our present studies, PCA results showed that tumor groups were independent of normal groups in each of the seven datasets (GSE8569, GSE21933, GSE33479, GSE33532, GSE40275, GSE62113, GSE74706). We identified 129 (91 up-regulated and 38 down-regulated) intersections of genes between GEO data and TCGA data. Chromosome mapping of consensus genes showed chromosomes 1 containing the greatest number of dysregulated genes in LUSC. Previously studies confirmed that the skewed X chromosome inactivation was associated with early development of lung cancer in females. The X chromosomal inactivation assay may be used to screen for females predisposed to malignancies including lung cancer^31^. Our results indicated that the dysregulation of FHL1 and FIGF on X chromosome may be associated with LUSC in females. On the other hand, Mosaic loss of the Y chromosome has a striking association with aging and cigarette smoking^32^. In our present study, that no differentially expressed gene was found in Y chromosome may be related to loss of Y chromosome gene.

We found that up-regulated genes were predominantly enriched in the activities of nuclear division, organelle fission, mitotic nuclear division, ATPase activity, microtubule binding and microtubule motor activity in LUSC. Meantime, down-regulated genes were enriched in humoral immune response, regulation of inflammatory response, regulation of cell growth, carboxylic acid binding, and response to transforming growth factor beta in LUSC. Previous studies showed that mitotic nuclear division is associated with cell proliferation, dysfunction of this process can lead to mitotic checkpoint failure and cause chromosome missegregation^33,34^. Microtubules function in the precise segregation of chromosomes during cell division, transport of cellular cargos, and positioning and movement of intracellular organelles^35^. Microtubule-binding drugs including the Vinca alkaloids and taxanes can suppress the dynamic instability of microtubules and induce apoptosis^36^. KEGG pathway enrichment analysis suggested significant enrichment in pathways including cell cycle and p53 signaling pathway. Our results indicated that the changes in biological processes, cellular components, molecular functions, and pathways may play critically important roles in the pathogenesis of LUSC. Protein-protein interaction network illustrated the overview of their functional connections. Module analysis of the PPI network suggested that protein serine/threonine kinase activity might be involved in LUSC development. Above are critical cellular processes for maintenance of cell homeostasis, dysregulation of these processes tends to promote carcinogenesis^37,38^. Our findings highlighted the probable importance of the regulation of these key biological behaviors by aberrantly expression in LUSC which warranted further investigations to confirm.

Previous studies confirmed that Enhancer of zeste homolog 2 (EZH2), which is a histone methyltransferase, can regulate gene expression by catalyzing tri-methylation of histone H3 at Lys 27 (H3K27me3)^39^. Behrens, C. *et al*. found that over expression of EZH2 was associated with tumor progression in lung cancer^40^. However, interestingly, it has been reported that EZH2 can also act as a tumor suppressor gene^41^. In our study, EZH2 was over-expressed and its higher expression predicted longer survival time for LUSC patients, indicating its potential tumor suppressor role in LUSC. ABCC5 functions have been regarded as a mediator of breast cancer skeletal metastasis. ABCC5 may be a potential therapeutic target for breast cancer bone metastasis^42^. KIF23 (Kinesin family member 23) is an important regulator of cellular cytokinesis, and it has been considered a tumor gene is glioma^43^. But its relationship with LUSC is largely unknown at present. A growing number of studies have confirmed that the lncRNAs-miRNAs-mRNAs regulation network functions in tumor pathogenesis and progression^38,44,45^. In our present study, ceRNA network found that PTHLH, EZH2, CEP55, CCNA2, PFN2, ABCC5, ANLN, UCK2, DSG2, GREM1, MYBL2, PITX1, CHEK1, KIF23 could be modulated by lncRNAs through corresponding miRNAs. This regulation network could provide us more knowledge of the sophisticated regulation patterns in LUSC. Strikingly, we also identified that 60 genes were statistically related to the overall survival rate. These overall survival-related genes were involved in the invasion of cells, proliferation of cells, respiratory of system tumor, differentiation of cells, and apoptosis. Previous studies reported that PART1 was associated with poor prognosis and tumor recurrence in stage I-III non-small cell lung cancer^46^. SFTA1P were regarded as a tumor suppressor. Down-regulation of SFTA1P may be associated with decreased TP53 expression^47^. LncRNA SNHG1 promoted non-small cell lung cancer progression^48^. In our present study, we found that over-expression of KC6, PART1, and SNHG1 were associated with poor prognosis in LUSC. However, lower expression of SFTA1P was associated with poor prognosis in LUSC.

In summary, our study analyzed the array-based and sequence-based data of LUSC supported by GEO and TCGA databases. We discovered a number of co-differentially expressed genes and important pathways in LUSC. Based on these genes, we performed a series of analyses, which may contribute to the finding of molecular mechanisms underlying the initiation and development of LUSC.

## Electronic supplementary material

Supplementary figures

Supplementary tables
