# Supplementary material for: Transcriptomic and functional network features of lung squamous cell carcinoma through integrative analysis of GEO and TCGA data
Source: Sci Rep. 2018 Oct 26;8:15834. doi: 10.1038/s41598-018-34160-w (PMC6203807; doi:10.1038/s41598-018-34160-w)
Supplement: Supplementary file 1 — Supplementary figures [file 41598_2018_34160_MOESM1_ESM.pdf]

Supplementary information:

Transcriptomic and functional network features of lung squamous cell carcinoma through integrative analysis of GEO and TCGA data

Yin Li, Jie Gu, Fengkai Xu, Qiaoliang Zhu, Di Ge[#] and Chunlai Lu[#]

Department of Thoracic Surgery, Zhongshan Hospital, Fudan University, Shanghai, P. R. China.

[#]Corresponding authors: Di Ge, M.D, Department of Thoracic Surgery, Zhongshan Hospital, Fudan University, 180 Fenglin Road, Shanghai, 200032, P.R.China, Tel. /Fax: +86 021 64041990-2559, E-mail address: ge.di@zs-hospital.sh.cn; Chunlai Lu, M.D, Department of Thoracic Surgery, Zhongshan Hospital, Fudan University, 180 Fenglin Road, Shanghai, 200032, P.R.China, Tel. /Fax: +86 021 64041990-2559, E-mail address: lu.chunlai@zs-hospital.sh.cn.

Running title: Transcriptomic and functional network features of lung squamous cell carcinoma

Figure legend:

Supplementary figure 1. Survival analysis for differentially expressed genes in LUSC. Survival curves showing 20 genes which were related to overall patient survival rate. P-value set for this analysis is less than 0.05.

Supplementary figure 2. Survival analysis for differentially expressed genes in LUSC. Survival curves showing 20 genes which were related to overall patient survival rate. P-value set for this analysis is less than 0.05.

Supplementary figure 3. Survival analysis showing four lncRNAs were associated with patients overall survival ($p < 0.05$).

Supplementary figure 4. Functional enrichment analysis for the 60 survival-related genes.

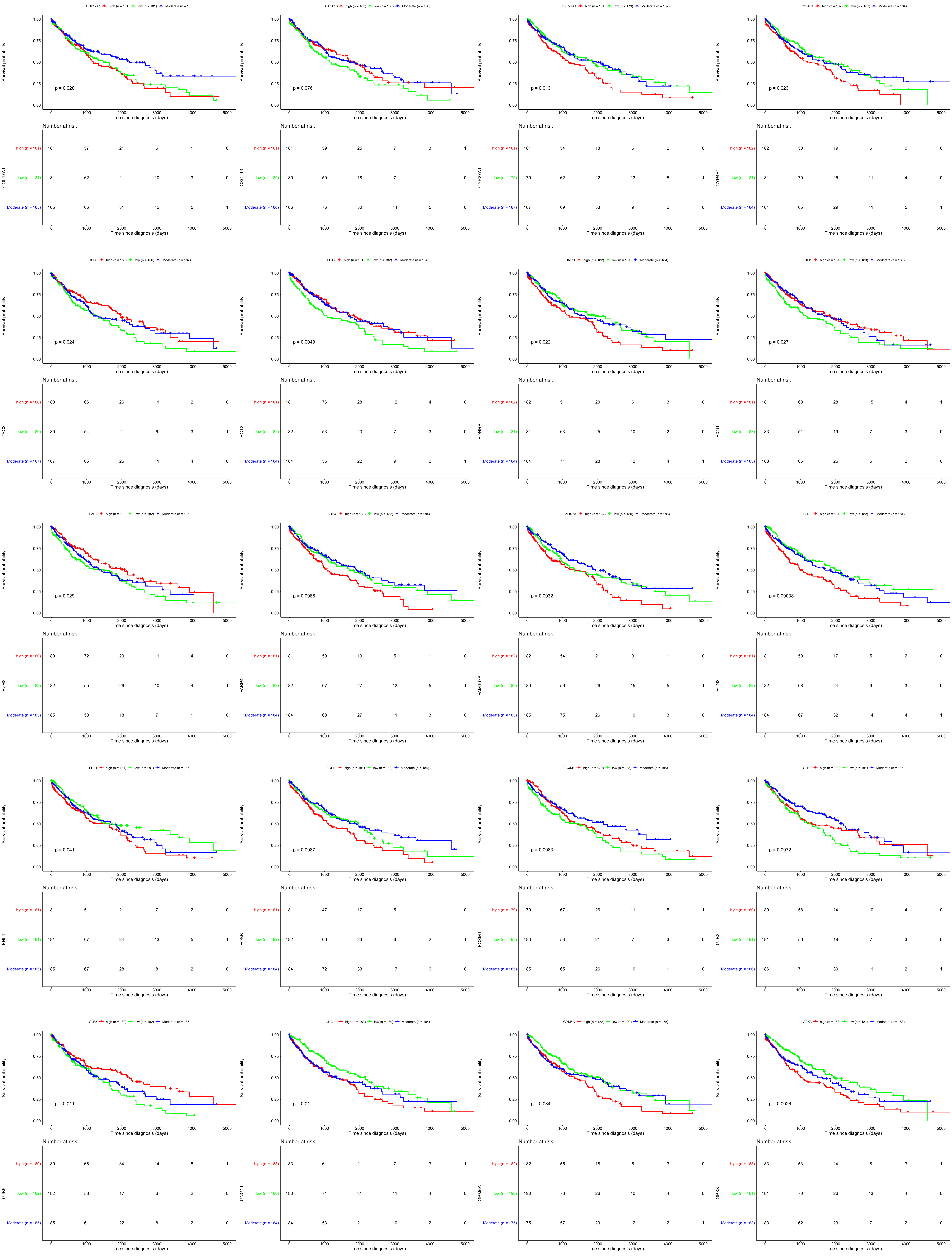
Survival probability
Time since diagnosis (days)
Number at risk
COL17A1 high (n = 181) low (n = 181) Moderate (n = 185)
p = 0.028
high (n = 181) 181 57 21 6 1 0
low (n = 181) 181 62 21 10 3 0
Moderate (n = 185) 185 66 31 12 5 1
CXCL13 high (n = 181) low (n = 180) Moderate (n = 186)
p = 0.076
high (n = 181) 181 59 25 7 3 1
low (n = 180) 180 50 18 7 1 0
Moderate (n = 186) 186 76 30 14 5 0
CYP27A1 high (n = 181) low (n = 179) Moderate (n = 187)
p = 0.013
high (n = 181) 181 54 18 6 2 0
low (n = 179) 179 62 22 13 5 1
Moderate (n = 187) 187 69 33 9 2 0
CYP4B1 high (n = 182) low (n = 181) Moderate (n = 184)
p = 0.023
high (n = 182) 182 50 19 6 0 0
low (n = 181) 181 70 25 11 4 0
Moderate (n = 184) 184 65 29 11 5 1
DSC3 high (n = 180) low (n = 180) Moderate (n = 187)
p = 0.024
high (n = 180) 180 66 26 11 2 0
low (n = 180) 180 54 21 6 3 1
Moderate (n = 187) 187 65 26 11 4 0
ECT2 high (n = 181) low (n = 182) Moderate (n = 184)
p = 0.0049
high (n = 181) 181 76 28 12 4 0
low (n = 182) 182 53 23 7 3 0
Moderate (n = 184) 184 56 22 9 2 1
EDNRB high (n = 182) low (n = 181) Moderate (n = 184)
p = 0.022
high (n = 182) 182 51 20 6 3 0
low (n = 181) 181 63 25 10 2 0
Moderate (n = 184) 184 71 28 12 4 1
EXO1 high (n = 181) low (n = 183) Moderate (n = 183)
p = 0.027
high (n = 181) 181 68 28 15 4 1
low (n = 183) 183 51 19 7 3 0
Moderate (n = 183) 183 66 26 6 2 0
EZH2 high (n = 180) low (n = 182) Moderate (n = 185)
p = 0.029
high (n = 180) 180 72 29 11 4 0
low (n = 182) 182 55 26 10 4 1
Moderate (n = 185) 185 58 18 7 1 0
FABP4 high (n = 181) low (n = 182) Moderate (n = 184)
p = 0.0086
high (n = 181) 181 50 19 5 1 0
low (n = 182) 182 67 27 12 5 1
Moderate (n = 184) 184 68 27 11 3 0
FAM107A high (n = 182) low (n = 180) Moderate (n = 185)
p = 0.0032
high (n = 182) 182 54 21 3 1 0
low (n = 180) 180 56 26 15 5 1
Moderate (n = 185) 185 75 26 10 3 0
FCN3 high (n = 181) low (n = 182) Moderate (n = 184)
p = 0.00038
high (n = 181) 181 50 17 5 2 0
low (n = 182) 182 68 24 9 3 0
Moderate (n = 184) 184 67 32 14 4 1
FHL1 high (n = 181) low (n = 181) Moderate (n = 185)
p = 0.041
high (n = 181) 181 51 21 7 2 0
low (n = 181) 181 67 24 13 5 1
Moderate (n = 185) 185 67 28 8 2 0
FOSB high (n = 181) low (n = 182) Moderate (n = 184)
p = 0.0087
high (n = 181) 181 47 17 5 1 0
low (n = 182) 182 66 23 6 2 1
Moderate (n = 184) 184 72 33 17 6 0
FOXM1 high (n = 179) low (n = 183) Moderate (n = 185)
p = 0.0083
high (n = 179) 179 67 26 11 5 1
low (n = 183) 183 53 21 7 3 0
Moderate (n = 185) 185 65 26 10 1 0
GJB2 high (n = 180) low (n = 181) Moderate (n = 186)
p = 0.0072
high (n = 180) 180 58 24 10 4 0
low (n = 181) 181 56 19 7 3 0
Moderate (n = 186) 186 71 30 11 2 1
GJB5 high (n = 180) low (n = 182) Moderate (n = 185)
p = 0.011
high (n = 180) 180 66 34 14 5 1
low (n = 182) 182 58 17 6 2 0
Moderate (n = 185) 185 61 22 8 2 0
GNG11 high (n = 183) low (n = 180) Moderate (n = 184)
p = 0.01
high (n = 183) 183 61 21 7 3 1
low (n = 180) 180 71 31 11 4 0
Moderate (n = 184) 184 53 21 10 2 0
GPM6A high (n = 182) low (n = 190) Moderate (n = 175)
p = 0.034
high (n = 182) 182 55 18 6 3 0
low (n = 190) 190 73 26 10 4 0
Moderate (n = 175) 175 57 29 12 2 1
GPX3 high (n = 183) low (n = 181) Moderate (n = 183)
p = 0.0026
high (n = 183) 183 53 24 8 3 1
low (n = 181) 181 70 26 13 4 0
Moderate (n = 183) 183 62 23 7 2 0

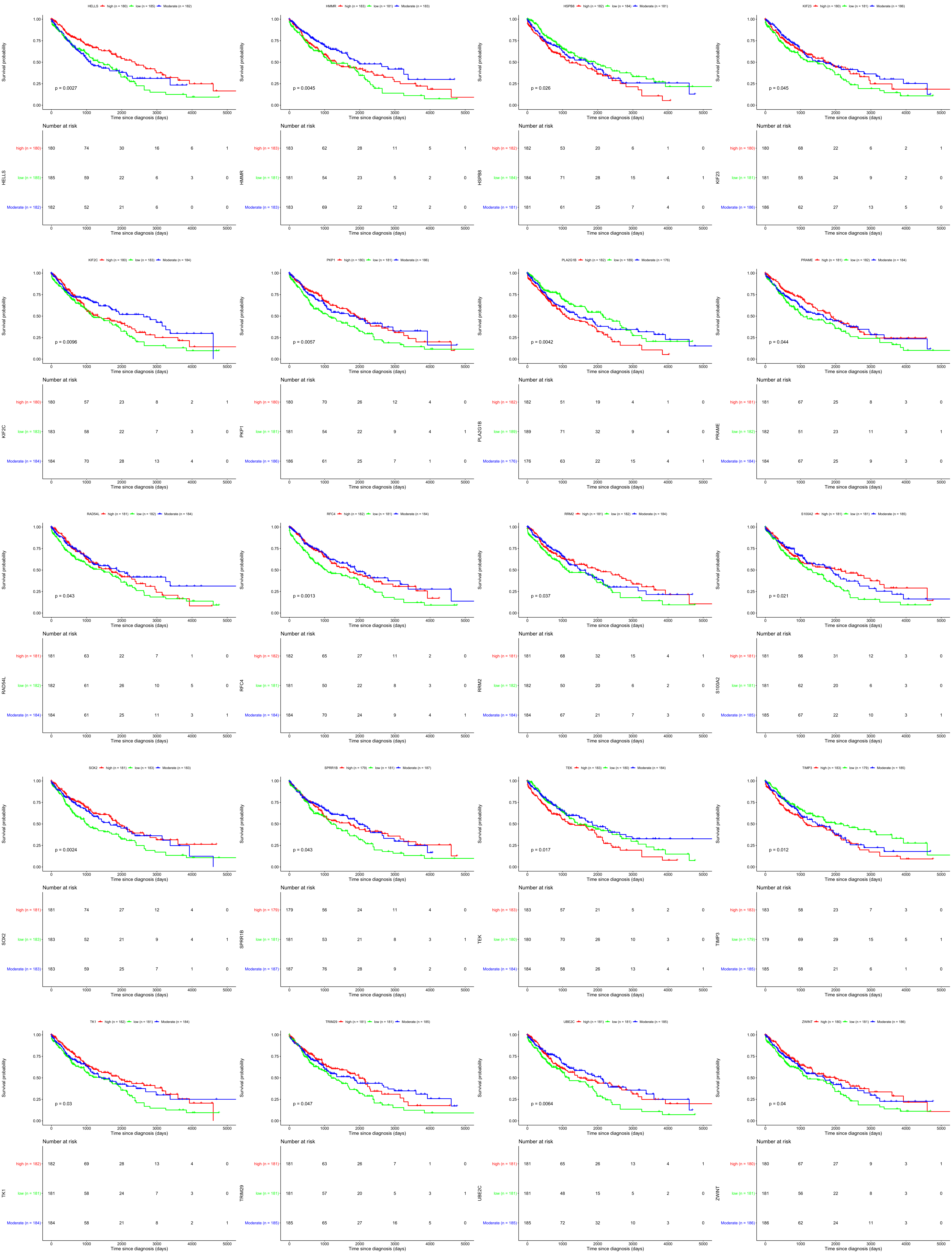
HELLS high (n = 180) low (n = 185) Moderate (n = 182)
p = 0.0027
HMMR high (n = 183) low (n = 181) Moderate (n = 183)
p = 0.0045
HSPB8 high (n = 182) low (n = 184) Moderate (n = 181)
p = 0.026
KIF23 high (n = 180) low (n = 181) Moderate (n = 186)
p = 0.045
KIF2C high (n = 180) low (n = 183) Moderate (n = 184)
p = 0.0096
PKP1 high (n = 180) low (n = 181) Moderate (n = 186)
p = 0.0057
PLA2G1B high (n = 182) low (n = 189) Moderate (n = 176)
p = 0.0042
PRAME high (n = 181) low (n = 182) Moderate (n = 184)
p = 0.044
RAD54L high (n = 181) low (n = 182) Moderate (n = 184)
p = 0.043
RFC4 high (n = 182) low (n = 181) Moderate (n = 184)
p = 0.0013
RRM2 high (n = 181) low (n = 182) Moderate (n = 184)
p = 0.037
S100A2 high (n = 181) low (n = 181) Moderate (n = 185)
p = 0.021
SOX2 high (n = 181) low (n = 183) Moderate (n = 183)
p = 0.0024
SPRR1B high (n = 179) low (n = 181) Moderate (n = 187)
p = 0.043
TEK high (n = 183) low (n = 180) Moderate (n = 184)
p = 0.017
TIMP3 high (n = 183) low (n = 179) Moderate (n = 185)
p = 0.012
TK1 high (n = 182) low (n = 181) Moderate (n = 184)
p = 0.03
TRIM29 high (n = 181) low (n = 181) Moderate (n = 185)
p = 0.047
UBE2C high (n = 181) low (n = 181) Moderate (n = 185)
p = 0.0064
ZWINT high (n = 180) low (n = 181) Moderate (n = 186)
p = 0.04
Survival probability
Time since diagnosis (days)
Number at risk

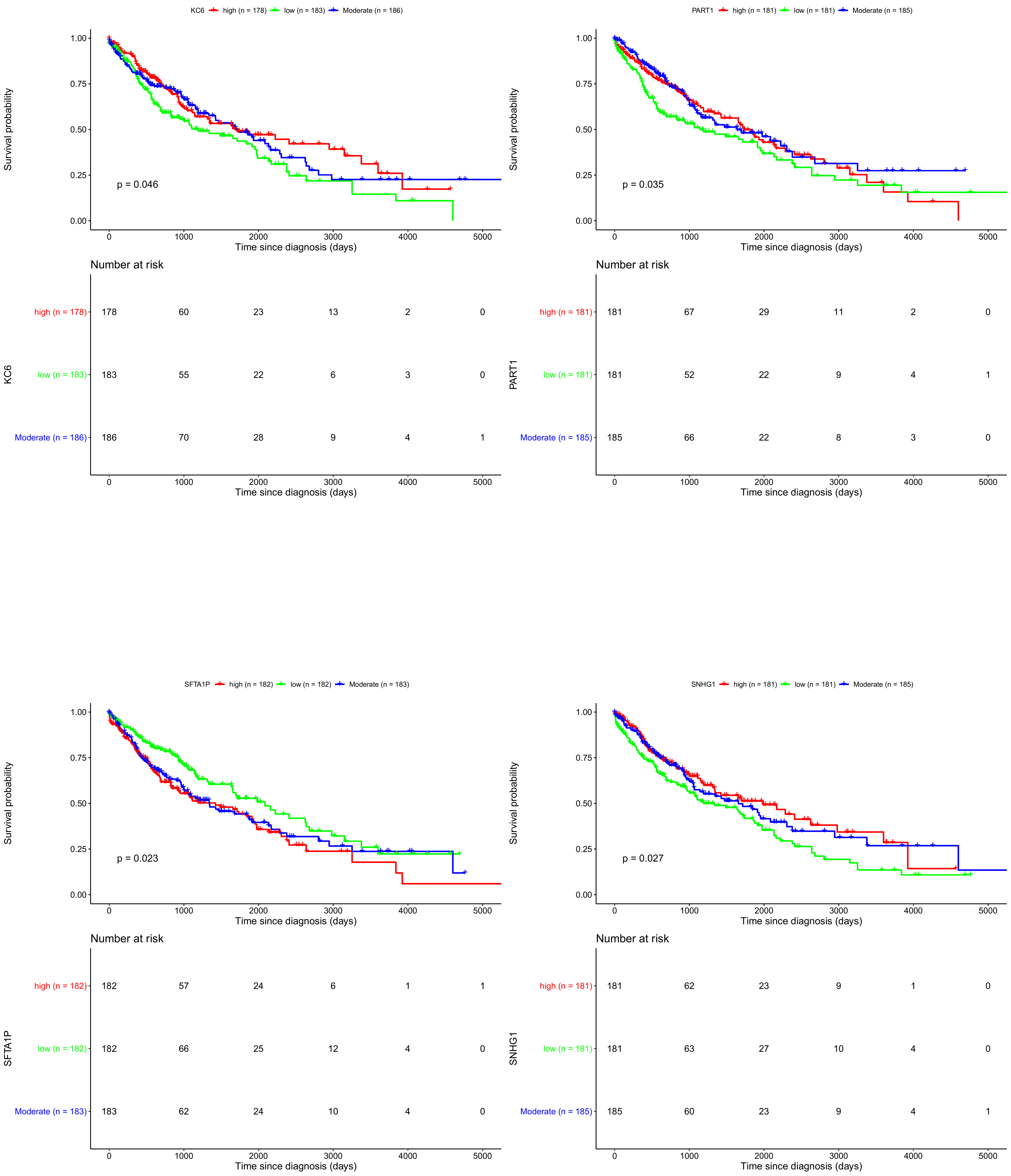

KC6 high (n = 178) low (n = 183) Moderate (n = 186)
Survival probability
1.00
0.75
0.50
0.25
0.00
p = 0.046
0 1000 2000 3000 4000 5000
Time since diagnosis (days)
Number at risk
KC6
high (n = 178) 178 60 23 13 2 0
low (n = 183) 183 55 22 6 3 0
Moderate (n = 186) 186 70 28 9 4 1
0 1000 2000 3000 4000 5000
Time since diagnosis (days)
PART1 high (n = 181) low (n = 181) Moderate (n = 185)
Survival probability
1.00
0.75
0.50
0.25
0.00
p = 0.035
0 1000 2000 3000 4000 5000
Time since diagnosis (days)
Number at risk
PART1
high (n = 181) 181 67 29 11 2 0
low (n = 181) 181 52 22 9 4 1
Moderate (n = 185) 185 66 22 8 3 0
0 1000 2000 3000 4000 5000
Time since diagnosis (days)
SFTA1P high (n = 182) low (n = 182) Moderate (n = 183)
Survival probability
1.00
0.75
0.50
0.25
0.00
p = 0.023
0 1000 2000 3000 4000 5000
Time since diagnosis (days)
Number at risk
SFTA1P
high (n = 182) 182 57 24 6 1 1
low (n = 182) 182 66 25 12 4 0
Moderate (n = 183) 183 62 24 10 4 0
0 1000 2000 3000 4000 5000
Time since diagnosis (days)
SNHG1 high (n = 181) low (n = 181) Moderate (n = 185)
Survival probability
1.00
0.75
0.50
0.25
0.00
p = 0.027
0 1000 2000 3000 4000 5000
Time since diagnosis (days)
Number at risk
SNHG1
high (n = 181) 181 62 23 9 1 0
low (n = 181) 181 63 27 10 4 0
Moderate (n = 185) 185 60 23 9 4 1
0 1000 2000 3000 4000 5000
Time since diagnosis (days)

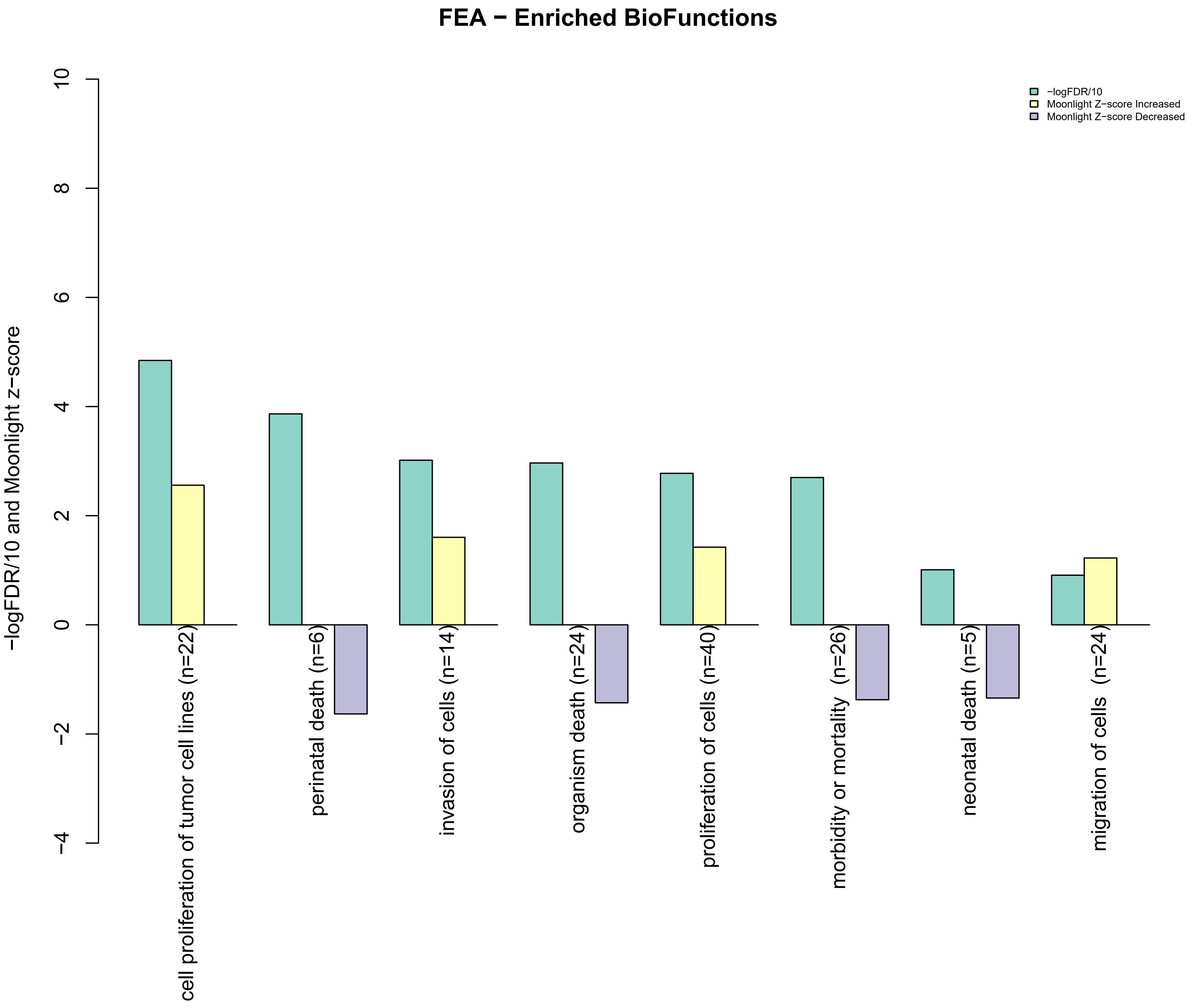
FEA − Enriched BioFunctions
−logFDR/10 and Moonlight z−score
10
8
6
4
2
0
−2
−4
−logFDR/10
Moonlight Z−score Increased
Moonlight Z−score Decreased
cell proliferation of tumor cell lines (n=22)
perinatal death (n=6)
invasion of cells (n=14)
organism death (n=24)
proliferation of cells (n=40)
morbidity or mortality (n=26)
neonatal death (n=5)
migration of cells (n=24)
